# Breast Cancer in the Setting of HIV

**DOI:** 10.4061/2011/925712

**Published:** 2011-05-25

**Authors:** Mitul Palan, Sami Shousha, Jonathan krell, Justin Stebbing

**Affiliations:** ^1^Department of Medical Oncology, The Hammersmith Hospitals NHS Trust, and Charing Cross Hospital, Imperial College Healthcare NHS Trust, Fulham Palace Road, London W6 8RF, UK; ^2^Department of Histopathology, and Charing Cross Hospital, Imperial College Healthcare NHS Trust, Fulham Palace Road, London W6 8RF, UK

## Abstract

Oncogenesis in immunocompromised patients occurs due to a number of factors including reduced immune surveillance or other viral pathogens. Breast cancer, unlike other non-AIDS-defining cancers, does not appear associated and has rarely been reported. We describe a case with evidence of immune reactivity around the tumor, but not in the tumor itself.

## 1. Introduction

Very few cases of breast cancer in HIV-infected women are recorded in the literature; approximately 46 HIV-infected women to this date have been recorded. According to the World Health Organization, approximately 33.4 million people (adults and children) in the world were living with HIV in 2008, the majority of which resided in sub-Saharan Africa [1]. A very wide range of cancers have been associated with an increased incidence in people living with HIV [2, 3]. As well as neoplasms such as Kaposi's sarcoma [4] and Non-Hodgkin's lymphoma [5], a focus of recent research has been the non-AIDS-defining cancers, that is, tumors other than Kaposi sarcoma, non-Hodgkin lymphoma, and cancer of the uterine cervix, in the setting of HIV. However, of the non-AIDS-defined cancers, breast cancer does not seem to increase in incidence within the HIV-infected population. This relationship between the two diseases has been noted worldwide, but few explanations have been forwarded as to the exact causative/noncausative relationship that the two may partake in. 

This paper looks to evaluate the relationship between the HIV and breast cancer, and we propose some theories as to why HIV infection may actually protect against breast tumor development.

## 2. Case Vignette

A 44-year-old HIV-positive lady presented with a right breast mass in the upper outer quadrant and was diagnosed with a triple negative (i.e., oestrogen receptor (ER), progesterone receptor (PR), and human epidermal growth factor 2 (HER2) negative), grade 3 invasive ductal carcinoma (IDC), 15 mm × 15 mm in size, with zero out of 3 sentinel nodes involved.

**Figure 1:**
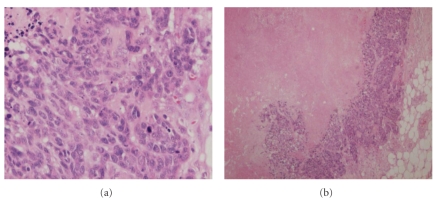
Low (a) and high (b) power images of our patient's invasive ductal carcinoma of the breast. Sections were stained with haematoxylin and eosin. (a) shows a large area of central tumor necrosis with a peripheral rim of viable tumor cells. (b) shows invasive tumor cells with high nuclear grade.

**Figure 2:**
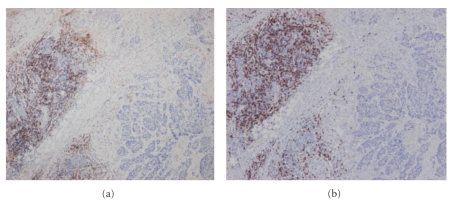
CD4 (a) and CD8 (b) staining of the tumor sections. Both cell populations were found adjacent to, but not infiltrating, the tumor.

She initially noticed a lump in her right breast, alongside skin and shape changes in the breast and nipple discharge. There was no history of oral contraceptive use, nor any family history of breast/ovarian cancer. Her CD4 count measured 450 cells/mm^3^ with an HIV-1 viral load of approximately 500 copies/mL. Mammography, ultrasound, and core biopsy of the right breast confirmed the presence of the tumor. Figures of her breast pathology may be seen below (Figures 1 and 2). CD4 and CD8 lymphocyte staining of the tissue sample showed significant lymphocytic infiltration adjacent to the tumor (but not within it).

The patient's treatment included a wide local excision of the lump with right axillary sentinel lymph node biopsy. The patient underwent 6 cycles of FEC75 chemotherapy requiring substantial dose reductions and G-CSF support, while remaining concurrently on HAART. A bone scan and a CT showed no sign of distant metastases. At the time of submission, she is completing her chemotherapy.

## 3. Discussion

While a number of neoplasms are well associated with HIV infection [4–7], non-AIDS-defining tumors [8] have more complex affiliations to HIV—some seem to also increase in incidence with infection, for example, lung cancer, myeloma, anal cancers [7, 9], while it is still debated whether others (e.g., breast cancer, head and neck cancer [10]) have an increased incidence due to HIV.

Few studies have delved into explaining the link between HIV and breast cancer. This is not particularly surprising however, as only 48 cases of breast cancer have been noted in HIV-positive people to date [11]. This number may be contested due to lack of case presentation (e.g., poorer socioeconomic groups/people of the third world less likely to seek medical care) or that women with HIV may have a reduced lifespan (and therefore are less likely to reach an age at which breast cancer may occur) [12]. A study in 1,416 HIV-positive Thai women, with an average age of 40.8 years over 5 years, found breast cancer to be the most prominent (9.5%) non-AIDS-defining tumors (42 cases of non-AIDS tumors overall) occurring [13]. It is important however to note though that the number of patients with breast cancer amounted to 4: approximately 0.28% of the percentage of women, a figure not too dissimilar to what one would expect in a female cohort such as this.

The majority of studies have found no difference in the incidence of breast cancer in HIV-positive women and the HIV-negative population [11]. An American study by Frisch et al. found the relative risk of developing breast cancer after HIV infection was 1.1, that is, HIV infection had little impact on breast cancer incidence [7], and other studies have supported this [9, 14]. In a sub-Saharan Tanzanian population, there was a small, yet statistically significant decrease in breast cancer incidence in the post-AIDS epidemic period (1983–1996), compared to the pre-AIDS period (1968–1983) [4]. Data from Rwanda additionally shows a low number of breast cancer cases despite a high HIV prevalence [15]. Reviews concur with these findings. For example, in South Africa, no trend has been observed [16].

People with HIV may develop cancer due to impaired immune surveillance, dysregulation of growth factors or cytokines, or imbalance between proliferation and differentiation [17]. As can be observed in our case, and in a number of other reported cases [18], the pathology of HIV-positive breast cancer patients is in stark contrast to noninfected women in the population. A reduction in immune function could be a reason why breast cancer prevalence remains low in patients with HIV [7, 19]. Pantanowitz investigated the pathology of HIV-positive breast cancer cases, finding that the immune response to these neoplasms was considerably less compared to that found in breast cancer in nonaffected women [3]. Immunosuppression is a large factor in the pathogenesis of many AIDS-related cancers [18, 20, 21]. HPV, associated with anal and cervical cancer (particularly in the immunocompromised), may induce oncogenesis by altering cell-cycle control—either deactivating tumor-suppressor genes or up regulating certain oncogenes [22]. There is continued debate whether HIV itself may cause dysregulation of cell proliferation by insertion of provirus nuclear material near/within human oncogenes [20].

The prognostic significance of stromal lymphocytic infiltration in breast cancer is disputed [23–26]. Previously, it has been linked to a poor prognosis [3, 27] (e.g., CD4- and CD8-T lymphocyte infiltration has been associated with high grade intraductal carcinomas [18]). This is not a relationship one would expect between the two as the immune system in nonimmunocompromised individuals plays a pivotal role in removing cancerous cells [18]. However, the control over cell proliferation here is slightly more complex. CD4- and CD8-T lymphocytes are implicated in acquired immune responses to cancer, but are tightly regulated by T regulatory cells [28]. Various studies have shown that T regulatory cells are found in abundance in neoplastic breast tissues, both in situ and in invasive carcinomas—their recruitment in intratumoral and peritumoral tissue may enable malignant cells to evade the host immune response, and as such may represent a marker of breast cancer progression [28, 29]. This may explain the absence of intratumoral lymphocytic infiltration, as seen in our patient (Figures 1 and 2). In HIV, however, the reduction in T cell count suggests that with a decreased number of T regulatory cells (along with some CD4- and CD8-T lymphocytes), the host is unable to restrict an immune response to cancer growth. This may indirectly benefit the patient from developing breast cancer, but perhaps not from other cancers [8]. Studies in posttransplant, non-HIV immunocompromised women (a similar subgroup to HIV-positive women) also support this proposition [16, 30]. 

HAART-induced restoration of immune function with treatment [31] may prevent initial breast tumorigenesis. Antiretrovirals such as ritonavir have been implicated in targeting both viral and tumorigenic proteins [32] that may play some part in breast cancer proliferation or tumor progression/resistance [33, 34]. These drugs may decrease the availability/activity of proangiogenic factors [5, 35], interfere with other oncogenic cytokine signalling pathways [31], or target intracellular mitogenic signalling pathways (which tumors use to proliferate [36, 37]). There is also some evidence to suggest that HIV viral proteins may induce tumor cell cytotoxicity and apoptosis [38]. Excessive fatty tissue contributes to breast cancer in postmenopausal women (as peripheral conversion of androgens to active oestradiol stimulates breast epithelial proliferation). The nutrient redistribution in HIV patients may have a role here [39–41], and the reduction in peripheral fat stores could reduce peripheral oestradiol conversion in these women. Overall, this seems an unlikely occurrence, especially in the few cases on breast cancer in HIV patients found.

## References

[2] Oluwole SF, Ali AO, Shafaee Z, Depaz HA (2005). Breast cancer in women with HIV/AIDS: report of five cases with a review of the literature. *Journal of Surgical Oncology*.

[3] Pantanowitz L, Connolly JL (2002). Pathology of the breast associated with HIV/AIDS. *Breast Journal*.

[4] Amir H, Kaaya EE, Kwesigabo G, Kiitinya JN (2000). Breast cancer before and during the AIDS epidemic in women and men: a study of Tanzanian Cancer Registry data 1968 to 1996. *Journal of the National Medical Association*.

[5] Stebbing J, Bower M (2003). What can oncologists learn from HIV?. *The Lancet Oncology*.

[6] Spina M, Nasti G, Simonelli C, Bertola G, Rossi C, Tirelli U (1994). Breast cancer in a women with HIV infection: a case report. *Annals of Oncology*.

[7] Frisch M, Biggar RJ, Engels EA, Goedert JJ (2001). Association of cancer with AIDS-related immunosuppression in adults. *Journal of the American Medical Association*.

[8] Stebbing J, Duru O, Bower M (2009). Non-AIDS-defining cancers. *Current Opinion in Infectious Diseases*.

[9] Chiao EY, Krown SE (2003). Update on non-acquired immunodeficiency syndrome-defining malignancies. *Current Opinion in Oncology*.

[10] Powles T, Robinson D, Stebbing J (2009). Highly active antiretroviral therapy and the incidence of non-AIDS-defining cancers in people with HIV infection. *Journal of Clinical Oncology*.

[11] Latif N, Rana F, Guthrie T (2011). Breast cancer and HIV in the era of highly active antiretroviral therapy: two case reports and review of the literature. *Breast Journal*.

[12] Pantanowitz L, Dezube BJ (2004). Reasons for a deficit of breast cancer among HIV-infected patients. *Journal of Clinical Oncology*.

[13] Kiertiburanakul S, Likhitpongwit S, Ratanasiri S, Sungkanuparph S (2007). Malignancies in HIV-infected Thai patients. *HIV Medicine*.

[14] Sarhan M, Depaz HA, Oluwole SFD (2010). Breast cancer in women with human immunodeficiency virus infection: pathological, clinical, and prognostic implications. *Journal of Women's Health*.

[15] Hurley J, Franco S, Gomez-Fernandez C (2001). Breast cancer and human immunodeficiency virus: a report of 20 cases. *Clinical Breast Cancer*.

[16] Guth AA (2003). Breast cancer and human immunodeficiency virus infection: issues for the 21st century. *Journal of Women’s Health*.

[17] Barbaro G (2007). Visceral fat as target of highly active antiretroviral therapy-associated metabolic syndrome. *Current Pharmaceutical Design*.

[18] DeNardo DG, Coussens LM (2007). Inflammation and breast cancer. Balancing immune response: crosstalk between adaptive and innate immune cells during breast cancer progression. *Breast Cancer Research : BCR*.

[19] Clifford G, Franceschi S (2007). Immunity, infection, and cancer. *The Lancet*.

[20] Kieff E (1998). Current perspectives on the molecular pathogenesis of virus-induced cancers in human immunodeficiency virus infection and acquired immunodeficiency syndrome. *Journal of the National Cancer Institute. Monographs*.

[21] Pantanowitz L, Schlecht HP, Dezube BJ (2006). The growing problem of non-AIDS-defining malignancies in HIV. *Current Opinion in Oncology*.

[22] Schecter WP (2001). Human immunodeficiency virus and malignancy: thoughts on viral Oncogenesis. *Archives of Surgery*.

[23] Hadden JW (1999). The immunology and immunotherapy of breast cancer: an update. *International Journal of Immunopharmacology*.

[24] Recchia F, Sica G, Candeloro G (2008). Maintenance immunotherapy in metastatic breast cancer. *Oncology Reports*.

[25] Rosen PP (2001). *Rosen’s Breast Pathology*.

[26] Sheu B-C, Kuo W-H, Chen R-J, Huang S-C, Chang K-J, Chow S-N (2008). Clinical significance of tumor-infiltrating lymphocytes in neoplastic progression and lymph node metastasis of human breast cancer. *Breast*.

[27] Murri AM, Hilmy M, Bell J (2008). The relationship between the systemic inflammatory response, tumor proliferative activity, T-lymphocytic and macrophage infiltration, microvessel density and survival in patients with primary operable breast cancer. *British Journal of Cancer*.

[28] Bates GJ, Fox SB, Han C (2006). Quantification of regulatory T cells enables the identification of high-risk breast cancer patients and those at risk of late relapse. *Journal of Clinical Oncology*.

[29] Gupta S, Joshi K, Wig JD, Arora SK (2007). Intratumoral FOXP3 expression in infiltrating breast carcinoma: its association with clinicopathologic parameters and angiogenesis. *Acta Oncologica*.

[30] Stewart T, Tsai SCJ, Grayson H, Henderson R, Opelz G (1995). Incidence of de-novo breast cancer in women chronically immunosuppressed after organ transplantation. *The Lancet*.

[31] Stebbing J, Bower M (2011). The anti-tumor effects of human immunodeficiency virus protease inhibitors: ready for real time? International journal of cancer. *Journal International du Cancer*.

[32] Huang PL, Sun Y, Chen HC, Kung HF, Huang PL, Lee-Huang S (1999). Proteolytic fragments of anti-HIV and anti-tumor proteins MAP30 and GAP31 are biologically active. *Biochemical and Biophysical Research Communications*.

[33] Bierman WFW, Scheffer GL, Schoonderwoerd A (2010). Protease inhibitors atazanavir, lopinavir and ritonavir are potent blockers, but poor substrates, of ABC transporters in a broad panel of ABC transporter-overexpressing cell lines. *The Journal of Antimicrobial Chemotherapy*.

[34] Gupta A, Zhang YI, Unadkat JD, Mao Q (2004). HIV protease inhibitors are inhibitors but not substrates of the human breast cancer resistance protein (BCRP/ABCG2). *The Journal of Pharmacology and Experimental Therapeutics*.

[35] Pore N, Gupta AK, Cerniglia GJ, Maity A (2006). HIV protease inhibitors decrease VEGF/HIF-1*α* expression and angiogenesis in glioblastoma cells. *Neoplasia*.

[36] Gupta AK, Cerniglia GJ, Mick R, McKenna WG, Muschel RJ (2005). HIV protease inhibitors block Akt signaling and radiosensitize tumor cells both in vitro and in vivo. *Cancer Research*.

[37] Srirangam A, Mitra R, Wang M (2006). Effects of HIV protease inhibitor ritonavir on Akt-regulated cell proliferation in breast cancer. *Clinical Cancer Research*.

[38] Muthumani K, Lambert VM, Sardesai NY (2009). Analysis of the potential for HIV-1 Vpr as an anti-cancer agent. *Current HIV Research*.

[39] Forrester JE, Spiegelman D, Tchetgen E, Knox TA, Gorbach SL (2002). Weight loss and body-composition changes in men and women infected with HIV. *American Journal of Clinical Nutrition*.

[40] Colecraft E (2008). HIV/AIDS: nutritional implications and impact on human development. *Proceedings of the Nutrition Society*.

[41] Leong PP, Mohammad R, Ibrahim N (2006). Phenotyping of lymphocytes expressing regulatory and effector markers in infiltrating ductal carcinoma of the breast. *Immunology Letters*.

